# Supplementary material for: How does it feel to run in minimalist and advanced footwear technology shoes: A qualitative study involving male recreational runners
Source: PLoS One. 2025 Dec 26;20(12):e0338743. doi: 10.1371/journal.pone.0338743 (PMC12742779; doi:10.1371/journal.pone.0338743)
Supplement: S1 Fig — The percentage (%) represents the number of participants who selected the reason as their first, second, or third reason from most (rank 1) to least (rank 3) important. The number of participants who selected the reason as their first, second, or third reason is provided in parentheses, respectively. (DOCX) [file pone.0338743.s001.docx]

**S1 Fig. Top three self-reported main reasons of participants (*n* = 18) for purchasing their current habitual running shoes.** The percentage (%) represents the number of participants who selected the reason as their first, second, or third reason from most (rank 1) to least (rank 3) important. The number of participants who selected the reason as their first, second, or third reason is provided in parentheses, respectively.
