# Supplementary material for: How does it feel to run in minimalist and advanced footwear technology shoes: A qualitative study involving male recreational runners
Source: PLoS One. 2025 Dec 26;20(12):e0338743. doi: 10.1371/journal.pone.0338743 (PMC12742779; doi:10.1371/journal.pone.0338743)
Supplement: Attachment — Submitted filename: PONE_Response_Final.docx [file pone.0338743.s004.docx]

**Manuscript Number**: PONE-D-25-14476

**Title:** How does it feel to run in minimalist and advanced footwear technology shoes: From anecdotal evidence to experimentation

**Journal:** PLOS ONE

We are greatly appreciative to the peer reviewer and editorial board for their time and valuable comments on our manuscript. We are pleased with the decision of the editorial board to reconsider our work for publication following revisions.

As requested, we have revised our manuscript according to the comments received and feel that these revisions have strengthened our manuscript. We provide below point-by-point responses to all comments. To facilitate the review process, changes to the original manuscript are formatted in red font except for areas where we have removed text. We trust that the reviewer and editorial board find our responses meet their expectations.

**Journal Requirements:**

When submitting your revision, we need you to address these additional requirements.

1. Please ensure that your manuscript meets PLOS ONE's style requirements, including those for file naming. The PLOS ONE style templates can be found at <https://journals.plos.org/plosone/s/file?id=wjVg/PLOSOne_formatting_sample_main_body.pdf> and <https://journals.plos.org/plosone/s/file?id=ba62/PLOSOne_formatting_sample_title_authors_affiliations.pdf>

**Response**: We have changed the formatting to align with the PLOS ONE style templates. These changes are not identified in red to ease readability.

2. Thank you for stating the following in the Competing Interests section:
Kim Hébert-Losier is a speaker for the Running Clinic, a continuing education organization that translates scientific evidence to healthcare professionals and the public. Internal university research funds were used to purchase all footwear used as part of this research.

Please confirm that this does not alter your adherence to all PLOS ONE policies on sharing data and materials, by including the following statement: "This does not alter our adherence to PLOS ONE policies on sharing data and materials.” (as detailed online in our guide for authors <http://journals.plos.org/plosone/s/competing-interests>). If there are restrictions on sharing of data and/or materials, please state these. Please note that we cannot proceed with consideration of your article until this information has been declared.

Please include your updated Competing Interests statement in your cover letter; we will change the online submission form on your behalf.

**Response**: We have added the requested statement to our disclosure.

3. We note that the grant information you provided in the ‘Funding Information’ and ‘Financial Disclosure’ sections do not match.

When you resubmit, please ensure that you provide the correct grant numbers for the awards you received for your study in the ‘Funding Information’ section.

**Response**: Thank you. Internal funds were used to purchase footwear used as part of this project, which did not derive from any specific grants or awards.

4. We note that you have indicated that there are restrictions to data sharing for this study. For studies involving human research participant data or other sensitive data, we encourage authors to share de-identified or anonymized data. However, when data cannot be publicly shared for ethical reasons, we allow authors to make their data sets available upon request. For information on unacceptable data access restrictions, please see <http://journals.plos.org/plosone/s/data-availability#loc-unacceptable-data-access-restrictions>.

Before we proceed with your manuscript, please address the following prompts:

a) If there are ethical or legal restrictions on sharing a de-identified data set, please explain them in detail (e.g., data contain potentially identifying or sensitive patient information, data are owned by a third-party organization, etc.) and who has imposed them (e.g., a Research Ethics Committee or Institutional Review Board, etc.). Please also provide contact information for a data access committee, ethics committee, or other institutional body to which data requests may be sent.

b) If there are no restrictions, please upload the minimal anonymized data set necessary to replicate your study findings to a stable, public repository and provide us with the relevant URLs, DOIs, or accession numbers. Please see <http://www.bmj.com/content/340/bmj.c181.long> for guidelines on how to de-identify and prepare clinical data for publication. For a list of recommended repositories, please see <https://journals.plos.org/plosone/s/recommended-repositories>. You also have the option of uploading the data as Supporting Information files, but we would recommend depositing data directly to a data repository if possible.

Please update your Data Availability statement in the submission form accordingly.

**Response:** We have amended our data availability statement to provide further information. Note that both reviewers stated we (the authors) made all data underlying the findings in our manuscript fully available.

***Data availability statement:*** *The data associated with the development of the themes discussed in this article are presented throughout the text as direct quotes from the participating runners. The transcribed datasets are not publicly available as the research participants did not provide consent for their data to be shared in online public repositories and the Ethics Committee deemed it would be ethically inappropriate to share the data openly. Data requests can be sent to the Human Research Ethics Committee of the University of Waikato (*[*humanethics@waikato.ac.nz*](mailto:humanethics@waikato.ac.nz)*) citing the approval number HREC(Health)2020#83. More detail on the Human Research Ethics Committee can be found at this link:* [*https://www.waikato.ac.nz/research/research-enterprise/ethics/human-research-ethics-committee/*](https://www.waikato.ac.nz/research/research-enterprise/ethics/human-research-ethics-committee/)*.*

5. Please provide a complete Data Availability Statement in the submission form, ensuring you include all necessary access information or a reason for why you are unable to make your data freely accessible. If your research concerns only data provided within your submission, please write "All data are in the manuscript and/or supporting information files" as your Data Availability Statement.

**Response:** This comment has been addressed above.

6. Please amend your manuscript to include your abstract after the title page.

**Response:** The abstract is present after the title page.

7. Please upload a copy of Figure 5, to which you refer in your text on page 21. If the figure is no longer to be included as part of the submission please remove all reference to it within the text.

**Response:** Thank you for noticing our mistake. Reference to “Figure 5” was inaccurate and has now been amended to refer to the correct Figure 4.

8. [Please remove all personal information, ensure that the data shared are in accordance with participant consent, and re-upload a fully anonymized data set.](http://track.editorialmanager.com/CL0/http:%2F%2Fwww.bmj.com%2Fcontent%2F340%2Fbmj.c181.long./1/010f01969ee3c551-5c0503fe-d584-40a8-94e6-ecba1e439b54-000000/9FVn2jq68kl8RtqoHOl--DdXOmDizN0kPdQEoIrkTmE=209)

[Note: spreadsheet columns with personal information must be removed and not hidden as all hidden columns will appear in the published file.](http://track.editorialmanager.com/CL0/http:%2F%2Fwww.bmj.com%2Fcontent%2F340%2Fbmj.c181.long./1/010f01969ee3c551-5c0503fe-d584-40a8-94e6-ecba1e439b54-000000/9FVn2jq68kl8RtqoHOl--DdXOmDizN0kPdQEoIrkTmE=209)

[Additional guidance on preparing raw data for publication can be found in our Data Policy (https://journals.plos.org/plosone/s/data-availability#loc-human-research-participant-data-and-other-sensitive-data) and in the following article: http://www.bmj.com/content/340/bmj.c181.long.](http://track.editorialmanager.com/CL0/http:%2F%2Fwww.bmj.com%2Fcontent%2F340%2Fbmj.c181.long./1/010f01969ee3c551-5c0503fe-d584-40a8-94e6-ecba1e439b54-000000/9FVn2jq68kl8RtqoHOl--DdXOmDizN0kPdQEoIrkTmE=209)

**Response:** This comment has been addressed above.

9. We notice that your supplementary figures are uploaded with the file type 'Figure'. Please amend the file type to 'Supporting Information'. Please ensure that each Supporting Information file has a legend listed in the manuscript after the references list.

**Response:** We have uploaded it as supporting information now.


[Note: HTML markup is below. Please do not edit.]

Reviewers' comments:

Reviewer's Responses to Questions

**Comments to the Author**

1. Is the manuscript technically sound, and do the data support the conclusions?

The manuscript must describe a technically sound piece of scientific research with data that supports the conclusions. Experiments must have been conducted rigorously, with appropriate controls, replication, and sample sizes. The conclusions must be drawn appropriately based on the data presented.

Reviewer #1: Yes

Reviewer #2: Yes

2. Has the statistical analysis been performed appropriately and rigorously?

Reviewer #1: Yes

Reviewer #2: Yes

3. Have the authors made all data underlying the findings in their manuscript fully available?

The [PLOS Data policy](http://track.editorialmanager.com/CL0/http:%2F%2Fwww.plosone.org%2Fstatic%2Fpolicies.action%23sharing/1/010f01969ee3c551-5c0503fe-d584-40a8-94e6-ecba1e439b54-000000/dFhMRhKr7KUXULxGsBzdYQCpTq_7EWGcL6ygQIcFZ5o=209) requires authors to make all data underlying the findings described in their manuscript fully available without restriction, with rare exception (please refer to the Data Availability Statement in the manuscript PDF file). The data should be provided as part of the manuscript or its supporting information, or deposited to a public repository. For example, in addition to summary statistics, the data points behind means, medians and variance measures should be available. If there are restrictions on publicly sharing data—e.g. participant privacy or use of data from a third party—those must be specified.

Reviewer #1: Yes

Reviewer #2: Yes

4. Is the manuscript presented in an intelligible fashion and written in standard English?

PLOS ONE does not copyedit accepted manuscripts, so the language in submitted articles must be clear, correct, and unambiguous. Any typographical or grammatical errors should be corrected at revision, so please note any specific errors here.

Reviewer #1: Yes

Reviewer #2: Yes

5. Review Comments to the Author

Please use the space provided to explain your answers to the questions above. You may also include additional comments for the author, including concerns about dual publication, research ethics, or publication ethics. (Please upload your review as an attachment if it exceeds 20,000 characters)

**Reviewer #1:** It is an interesting study, based on questionnaire method. I specifically admire the originality of this work, regarding the comfort level of shoes. I consider that sections Introduction and Discussions gets the reader familiarized with the topic.

**Response**: Thank you for the positive comments on our work.

This study employed a qualitative approach to explore male recreational runners' subjective experiences with minimalist running shoes (FLAT) and advanced footwear technology (AFT) shoes, revealing complex perceptions regarding comfort, performance, biomechanical changes, and injury risk. The findings indicate that runners' evaluations of footwear are a dynamic, iterative process influenced by novelty, familiarity, and actual running experience. OWN shoes were preferred due to familiarity, while AFT and FLAT elicited mixed reactions due to their extreme designs. The study highlights the need for runners to balance comfort, performance, and injury risk when selecting footwear and recommends a gradual transition to new shoe models. However, the research has limitations, including a restricted sample, limited shoe variety, and methodological constraints in data collection. Detailed comments from the reviewer are as follows:

1.Limited sample diversity. Recommend including female runners or explicitly stating conclusions apply only to male populations.

**Response:** We agree that the findings of the present project likely do not extend to other running populations. Given the novelty of this project, we considered it suitable to maintain a focused research question within a specific population of male adult recreational runners. Our limitations section explicitly refers to limitations of the study involving only male runners, limiting generalisability of findings to recreational female runners and other running populations. We also suggest that future research expand sample diversity.

We also note that comments #2 and #3 from the reviewer relate to limitations which are addressed in the text below.

***Discussion:*** *Several limitations should be acknowledged. Our sample consisted solely of male recreational runners, limiting the generalisability of findings to female recreational runners and other running populations (e.g., professional athletes or different age groups). Additionally, we examined only one model of AFT and minimalist shoes, which may not represent the full spectrum of footwear designs available [9]. Furthermore, each trial involved a 1.5 km run, which may not reflect real experiences during long-distance running or daily training. Future studies should extend the running distance and expand sample diversity, exploring broader demographics and a wider range of shoe models and brands to build on these findings.*

2.Restricted shoe representation. Testing only one AFT and FLAT model limits generalizability. Suggest adding other models within each category to enhance external validity.

**Response:** Our limitation section (excerpt provided in response to comment #1) outlines the limitations of the study involving only one model of AFT and minimalist shoes, and suggests including more brands and models in future studies.

3.Insufficient running distance. 1.5 km may not adequately reflect long-term adaptation. Recommend extending single-run distance or adding multiple trials.

**Response:** The reviewer makes a valid point. We have added these considerations in our limitation section.

***Discussion:*** *Furthermore, each trial involved a 1.5 km run, which may not reflect real experiences during long-distance running or daily training. Future studies should extend the running distance and expand sample diversity, exploring broader demographics and a wider range of shoe models and brands to build on these findings.*

4.Unclear pace control. "Comfortable pace" and "race pace" lack quantitative definitions. Should supplement with pace ranges or heart rate/RPE data to standardize intensity.

**Response:** Participants wore a Garmin 245 Music watch (Garmin Ltd., Olathe, Kansas) during their trials to monitor their 1.1 km and 400 m running times, enabling us to calculate running speeds. We have added this information in the methods section and report their running speeds in Table 1. We specify in the Table notes that there were no significant differences in 1.1 km (*P* = 0.447) and 400 m (*P* = 0.071) speed between shoes based on repeated measures analysis of variance

***Materials and methods:*** *Participants wore a Garmin 245 Music watch (Garmin Ltd., Olathe, Kansas) during their running efforts to monitor their 1.1 km and 400 m running times, enabling us to calculate running speeds (Table 1).*

5.Lack of objective measures. Over-reliance on subjective interviews. Recommend incorporating biomechanical (e.g., gait analysis) or physiological (e.g., metabolic cost) data to validate perceptions.

**Response:** Monitoring biomechanical and physiological data extends beyond the aims of the present study, which were to explore recreational runners’ perceptions of novel shoes in a real-world setting. We explore their perceptions using both subjective (interview) and objective (visual analogue scale and ranking) methods. Hence, we deem our approach appropriate to answer our research question. However, we agree that these perspectives warrant investigation in future research. We have included specific mention of this aspect in our discussion.

***Discussion:*** *We are unable to confirm whether the perceived biomechanical changes runners felt running in novel shoes were in fact linked with actual biomechanical changes, especially considering how self-perceived foot strike patterns demonstrate low accuracy (42.7%) in endurance runners [1]. Future research should seek to corroborate subjective perceptions of runners to biomechanical measures.*

6.Interview method limitations. Neo-positivist approach may constrain depth. Suggest semi-structured or phenomenological interviews for richer subjective data capture.

**Response**: We agree that this is a limitation which should be considered when interpreting the results of our findings. This suggestion is articulated in the discussion of the manuscript, with specific recommendations for future research, as follows.

***Discussion****: While an interpretive phenomenological framework (interpretivist epistemology) was used for data analysis, data collection followed a neo-positivist approach (constructivist ontology) to minimise researcher influence on participant responses. This hybrid methodology may have influenced the depth of interpretative engagement.* *Future research could benefit from a more constructivist interviewing approach, such as semi-structured or phenomenological interviews, to explore runners’ subjective experiences in greater depth*

7.Inadequate controls for order effects. Using OWN shoes consistently as baseline may introduce bias. Recommend full randomization of all shoe testing sequences.

**Response**: Having own shoe was an active choice and selected for it to act as a baseline comparator to novel shoes. Doing so is justified in the manuscript, and we respectfully rebut this point as we believe this approach is justifiable, and it is not possible to change our approach without repeating the study.

8.Unstandardized environmental factors. Outdoor trials affected by weather/terrain. Should document conditions (temperature, surface) and discuss potential impacts.

**Response**: We agree that outdoor trials, although conferring high ecological validity, reduces the ability for standardisation of environmental factors. We have included details of climatic conditions during testing in the methods section (under the heading ‘protocol’):

***Materials and methods:*** *The average atmospheric temperature and humidity during testing sessions was 12.8 ± 3.3°C and 82.5 ± 4.5%, with an average wind velocity of 15.1 ± 3.0 km/h..*

We did not specifically control for climatic/environmental factors, and there are a range of microclimatic variables, such as surface temperature, which were not captured. Therefore the following statements have been added in the limitations section of our discussion.

***Discussion:*** *The trials were conducted outdoors, which confers high ecological validity, but lacks environmental standardisation. Variables such as ambient temperature, relative humidity, solar radiation, and wind velocity may have influenced runners’ comfort perceptions. For instance, elevated temperatures can increase in-shoe heat and moisture, potentially affecting thermal comfort [2]. Ambient temperatures also influence footwear properties and material characteristics [3, 4], which can in turn influence runners’ comfort perceptions. Although all participants ran on the same surface and followed the same route, we acknowledge that environmental variability may have contributed to subjective comfort ratings.*

9.Insufficient data analysis transparency. Need detailed description of inter-coder reliability checks (e.g., Kappa values) in thematic analysis to enhance credibility.

**Response**: We thank the reviewer for their thoughts, and we agree that consistency and agreement between coders are important. However, as the reviewer may be aware, evaluating intercoder reliability explicitly is somewhat controversial in qualitative research [5]. We hold the perspective that these processes go against the interpretative nature of qualitative data analysis. We now further highlight that we implemented regular inter-coder checks in our coding process, and coded data following systematic processes described in the literature.

***Materials and methods****: Data were coded using a systematic process, where statements from runners were analysed and categorised into themes representing the phenomenon of interests [6].*

***Materials and methods:*** *As participants did not directly validate the findings due to time constraints, participant availability, and to limit recall bias; the research team engaged in iterative theme validation to ensure alignment with participant narratives and inter-coder checks, minimising researcher interpretation bias of underlying concepts that were implied in participant responses. Themes and codes were reviewed by three members of the research team (BP, KHL, HK) at regular time points in a collaborative process to ensure accuracy, with participant quotes (abbreviated ‘P’) providing evidence and context.*

10.Missing participant validation. Recommend member checking by returning preliminary findings to participants to ensure interpretive accuracy.

**Response**: As we were interested in participants acute experiences running in novel footwear, interview transcripts were not returned to participants to avoid recall bias and contamination of acute perceptions. We have added the following specifications in our manuscript.

***Materials and methods****: Interviews were voice recorded in real-time to enhance accuracy and limit recall bias [7].*

***Materials and methods****: As the interviews were conducted immediately before and after running in the novel shoes, returning the transcribed interviews would be susceptible to recall bias due to the extended timeframe [7].*

11.Unreported blinding effectiveness. Despite painted shoes, should assess if participants identified brands/types through other cues.

**Response**: Adding this information is very relevant, thank you for noticing. We had also considered this in the study design, however, accidentally omitted this information. We have now incorporated this information in the results section now.

***Results****: Of the 18 runners, five correctly identified the brand of the AFT (27.8%) and two the brand of the FLAT (11.1%). No runner knew the specific model of these shoes.*

12.Long-term adaptations unexplored. Single trial cannot reflect prolonged use effects. Recommend adding runners' perspectives on transition periods or extended usage.

**Response**: We now have a dedicated section that goes into transition periods in the discussion, aligning with this comment and those raised by Reviewer #2.

***Discussion:*** *Furthermore, it is challenging to unequivocally recommend or establish transitioning timelines for novel footwear from existing literature [8], with a minimum of 4 to 8 weeks of gradual transitioning to minimalist shoes recommended for general musculoskeletal adaptations [8]. As AFT are more like traditional shoes in their design and comfort levels than minimalist shoes [9], the habituation period to AFT compared to minimalist shoes may be quicker for runners used to running in more traditional footwear. Nonetheless, the time and training requirements for habituation and adaptation to novel shoes remain relatively unknown and are likely specific to individuals and shoes.*

**Reviewer #2:** This study conducted real-world outdoor experiments with 18 male recreational runners, comparing their subjective perceptions and running experiences with minimalist shoes and advanced footwear technology (AFT) shoes. It highlights the trade-offs runners make between comfort, performance, and injury risk when selecting footwear. The study contributes to a deeper understanding of runners' footwear perceptions and offers practical insights for shoe design and selection. However, its main limitations include a homogeneous sample, limited shoe models, and reliance on subjective data. The detailed reviewer comments are as follows:

1. The title mentions “from anecdotal evidence to experiment,” but neither the abstract nor the main text clearly references the specific anecdotal evidence or how it is compared to experimental results. It is recommended to supplement relevant background in the introduction or discussion section to enhance coherence between the title and the content.

**Response:** We have removed the mention of “from anecdotal evidence to experiment” in the title to avoid ambiguity. In additional, we now explicitly state in the title that male recreational runners were involved to clarify this focus.

***Title:*** *How does it feel to run in minimalist and advanced footwear technology shoes: A mixed-methods study involving male recreational runners*

2. The study includes only male runners, without involving female runners or other populations (e.g., professional athletes or different age groups). It is advised to explicitly discuss gender and population limitations in the limitations section and suggest expanding sample diversity in future research.

**Response:** Our limitation section explicitly refers to limitations of the study involving only male runners, limiting generalisability of findings to recreational female runners and other running populations. We also suggest that future research expand sample diversity.

***Discussion:*** *Several limitations should be acknowledged. Our sample consisted solely of male recreational runners, limiting the generalisability of findings to female recreational runners and other running populations (e.g., professional athletes or different age groups). Additionally, we examined only one model of AFT and minimalist shoes, which may not represent the full spectrum of footwear designs available [9]. Furthermore, each trial involved a 1.5 km run, which may not reflect real experiences during long-distance running or daily training. Future studies should extend the running distance and expand sample diversity, exploring broader demographics and a wider range of shoe models and brands to build on these findings.*

In additional to the above, we now explicitly state in the title that male recreational runners were involved to clarify this focus.

***Title:*** *How does it feel to run in minimalist and advanced footwear technology shoes: A mixed-methods study involving male recreational runners*

3. The study used only one minimalist shoe and one AFT shoe, which may not represent the diversity of similar footwear. It is recommended to emphasize this limitation in the discussion section and suggest including more brands or models in future studies.

**Response:** We agree that this is a limitation of our study, and the limitations section of our manuscript explicitly acknowledges the limitations of the study involving only one model of AFT and minimalist shoes, and suggests including more brands and models in future studies, as indicated in the previous response to the reviewer.

4. The study relies on semi-structured interviews and subjective feedback, which may introduce recall bias or social desirability bias. It is suggested to clarify in the methods section how these biases were mitigated (e.g., through real-time recording or anonymous feedback).

**Response:** We thank the reviewer for highlighting the potential for recall and social desirability biases. We now further clarify in the revised Methods section how these biases were mitigated.

To limit recall bias, interviews were conducted immediately before and after running trials. Interviews were voice recorded in real-time to enhance accuracy and limit recall bias. Interviews were not returned to runners as doing so would enhance recall bias and any modifications would not reflect runners’ immediate experiences running in novel footwear.

To mitigate social desirability bias, all interviews were conducted in a one-on-one setting and participants were assured their answers would remain confidential. Furthermore, the interviewer used a neo-positivist’ neutral-questioning approach to mitigate social desirability bias, alongside developing a rapport prior to study commencement to enhance trust.

***Materials and methods:*** *Participants had one-on-one interviews of one-to-three minutes in duration immediately before and after the running trials to discuss their perceptions regarding each shoe, limiting recall bias [7].*

***Materials and methods:*** *The same male researcher who was a sport scientist, runner, and laboratory technician conducted all interviews (SF, MSc) using a ‘neo-positivist’ neutral-questioning approach, in which the interviewer remained neutral to participants’ responses to mitigate social desirability bias [10], alongside developing a rapport prior to study commencement to enhance trust.*

***Materials and methods:*** *To reduce social desirability bias, the interviews were conducted in a one-on-one setting and participants were assured their answers would remain confidential [11]. Interviews were voice recorded in real-time to enhance accuracy and limit recall bias [7].*

***Materials and methods:*** *As the interviews were conducted immediately before and after running in the novel shoes, returning the transcribed interviews would be susceptible to recall bias due to the extended timeframe [7] and any change would likely not reflect runners’ immediate experiences.*

5. Each trial involved only a 1.5 km run, which may not reflect real experiences during long-distance running or daily training. It is advised to acknowledge this limitation in the discussion and recommend extending the running distance or increasing the number of trials in future studies.

**Response:** The reviewer makes a valid point. We have added these considerations in our limitation section.

***Discussion:*** *Furthermore, each trial involved a 1.5 km run, which may not reflect real experiences during long-distance running or daily training. Future studies should extend the running distance and expand sample diversity, exploring broader demographics and a wider range of shoe models and brands to build on these findings.*

6. Although a six-phase thematic analysis was mentioned, there was no detailed explanation of how coding reliability and validity were ensured (e.g., inter-coder reliability checks). It is suggested to add specific steps or cite relevant methodological literature.

**Response**: We thank the reviewer for their thoughts, and we agree that consistency and agreement between coders are important. However, as the reviewer may be aware, evaluating intercoder reliability explicitly is somewhat controversial in qualitative research [5], and goes against the interpretative nature of qualitative data. We now further highlight that we implemented regular inter-coder checks in our coding process, and coded data following systematic processes described in the literature.

***Materials and methods****: Data were coded using a systematic process, where statements from runners were analysed and categorised into themes representing the phenomenon of interests [6].*

***Materials and methods:*** *As participants did not directly validate the findings due to time constraints, participant availability, and to limit recall bias; the research team engaged in iterative theme validation to ensure alignment with participant narratives and inter-coder checks, minimising researcher interpretation bias of underlying concepts that were implied in participant responses. Themes and codes were reviewed by three members of the research team (BP, KHL, HK) at regular time points in a collaborative process to ensure accuracy, with participant quotes (abbreviated ‘P’) providing evidence and context.*

7. The results section is relatively long, but the discussion lacks in-depth analysis of certain findings (e.g., the conflict between “familiarity” and “novelty”). It is suggested to further explore the mechanisms or theoretical implications of these phenomena by referencing existing literature.

**Response**: We appreciate the reviewer raising this area worthy of discussion. We have added a section on this topic during the revision of our work.

***Discussion:*** *The data drawn from the interviews highlight the conflict between familiarity and novelty in our runners’ preference, with familiarity shown elsewhere to evoke “liking” and novelty to evoke “interest” [12]. Runners tended to perceive familiar shoes (e.g., their own) more favourably likely due to an increased processing fluency – a phenomenon where familiar stimuli are easier to mentally process and therefore feel more comfortable or preferable [13]. At the same time, novelty seeking can drive preference due to its association with excitement, curiosity, and stimulation [14].*

***Discussion****: The more positive perceptions towards novel shoes after participants ran with them reflect the mere-repeated-exposure paradigm, where repeated exposure enhances the positive affect of individuals towards a stimulus object [15].*

8. The terms “AFT” and “VP4” are used interchangeably (e.g., in tables), which may cause confusion. It is recommended to consistently use either “AFT” or the full name throughout the text.

**Response**: We thank the reviewer for spotting our inconsistency. We have amended to consistently use AFT throughout.

9. Although ethical approval was mentioned, it was not specified whether participants signed written consent forms or how their privacy was protected. It is advised to provide these details in the methods section.

**Response**: We thank the reviewer for noticing our oversight. We have added the following statement to our methods “All participants signed an informed consent document prior to participating, which assured their data would remain confidential.”

10. The conclusion mentions “gradual adaptation to new shoe types,” but does not provide specific transition plans (e.g., time, intensity). It is suggested to include practical recommendations based on the study results in the discussion or conclusion section.

**Response**: We thank the reviewer for their comments. Despite the results pointing to the need for gradual adaptation, providing specific recommendations based on the perceptions of runners is unfeasible given the recommendations would derive from an acute experience of running in novel shoes for 1.5 km. Furthermore, the appropriate posology for transition is relatively unknown and likely shoe and individual specific. We have added the following section in our discussion to address this topic.

***Discussion:*** *Furthermore, it is challenging to unequivocally recommend or establish transitioning timelines for novel footwear from existing literature [8], with a minimum of 4 to 8 weeks of gradual transitioning to minimalist shoes recommended for general musculoskeletal adaptations [8]. As AFT are more like traditional shoes in their design and comfort levels than minimalist shoes [9], the habituation period to AFT compared to minimalist shoes may be quicker for runners used to running in more traditional footwear. Nonetheless, the time and training requirements for habituation and adaptation to novel shoes remain relatively unknown and are likely specific to individuals and shoes.*

11. It may be beneficial to compare these findings with the data-driven gait recognition approach proposed by Xu et al., to explore the complementarity between subjective feedback and objective identification. Such a comparison could enrich the understanding of how footwear choices influence gait and provide a theoretical foundation for future multidimensional analyses that integrate both qualitative and quantitative methods. (A new method proposed for realizing human gait pattern recognition: Inspirations for the application of sports and clinical gait analysis (<https://doi.org/10.1016/j.gaitpost.2023.10.019)>)

**Response**: We thank the reviewer for their comment. The paper reference (<https://doi.org/10.1016/j.gaitpost.2023.10.019>) examines biomechanical gait features for gait pattern recognition and it does not address subjective feedback for objective identification as the reviewer suggests. We are finding it challenging to integrate this recommendation as the felt biomechanical changes from runners running in the novel shoes were not actually measured (e.g., GRF relating to jarring or impact sensations). We know from scientific literature that self-reported foot strike patterns from runners, for instance, are not an accurate representation of actual foot strike patterns [1]. We also know that runners can alter gait patterns without specific instructions to achieve a certain goal, like reducing the peak tibial acceleration during running [16]. The following statement has been added in the discussion.

***Discussion:*** *We are unable to confirm whether the perceived biomechanical changes runners felt running in novel shoes were in fact linked with actual biomechanical changes, which is important to consider in the context of existing evidence demonstrating low accuracy of runners’ self-reported gait parameters, for example, foot strike patterns [1]. Future research should seek to corroborate subjective perceptions of runners to biomechanical measures..*

6. PLOS authors have the option to publish the peer review history of their article ([what does this mean?](https://track.editorialmanager.com/CL0/https:%2F%2Fjournals.plos.org%2Fplosone%2Fs%2Feditorial-and-peer-review-process%23loc-peer-review-history/1/010f01969ee3c551-5c0503fe-d584-40a8-94e6-ecba1e439b54-000000/11KTBPXM0HEG5oEj_O1jmRPBEPCzlKJUMT1sZFIhMD0=209)). If published, this will include your full peer review and any attached files.


If you choose “no”, your identity will remain anonymous but your review may still be made public.


**Do you want your identity to be public for this peer review?** For information about this choice, including consent withdrawal, please see our [Privacy Policy](https://track.editorialmanager.com/CL0/https:%2F%2Fwww.plos.org%2Fprivacy-policy/1/010f01969ee3c551-5c0503fe-d584-40a8-94e6-ecba1e439b54-000000/PX84z8G8eMXSBT7P_1TzRXs48F83emdwPDEuVhydp28=209).

Reviewer #1: **Yes:** Diana Ciubotariu

Reviewer #2: No

[NOTE: If reviewer comments were submitted as an attachment file, they will be attached to this email and accessible via the submission site. Please log into your account, locate the manuscript record, and check for the action link "View Attachments". If this link does not appear, there are no attachment files.]

While revising your submission, please upload your figure files to the Preflight Analysis and Conversion Engine (PACE) digital diagnostic tool, [https://pacev2.apexcovantage.com/](https://track.editorialmanager.com/CL0/https:%2F%2Fpacev2.apexcovantage.com%2F/1/010f01969ee3c551-5c0503fe-d584-40a8-94e6-ecba1e439b54-000000/3Ls0fPWqB55YqgjP-_-Va-pbww2pX3K3hxmZvfmS3S8=209). PACE helps ensure that figures meet PLOS requirements. To use PACE, you must first register as a user. Registration is free. Then, login and navigate to the UPLOAD tab, where you will find detailed instructions on how to use the tool. If you encounter any issues or have any questions when using PACE, please email PLOS at [figures@plos.org](mailto:figures@plos.org). Please note that Supporting Information files do not need this step.

**References (cited in the response document)**

1.	Vincent HK, Coffey K, Villasuso A, Vincent KR, Sharififar S, Pezzullo L, Nixon RM. Accuracy of self-reported foot strike pattern detection among endurance runners. Frontiers in Sports and Active Living. 2024;6:1491486. doi: 10.3389/fspor.2024.1491486

2.	West AM, Schönfisch D, Picard A, Tarrier J, Hodder S, Havenith G. Shoe microclimate: An objective characterisation and subjective evaluation. Applied Ergonomics. 2019;78:1-12. doi: 10.1016/j.apergo.2019.01.010

3.	Kinoshita H, Bates BT. The effect of environmental temperature on the properties of running shoes. Journal of Applied Biomechanics. 1996;12(2):258-68. doi: 10.1123/jab.12.2.258

4.	Shariatmadari MR, English R, Rothwell G. Effects of temperature on the material characteristics of midsole and insole footwear foams subject to quasi-static compressive and shear force loading. Materials & Design. 2012;37:543-59. doi: 10.1016/j.matdes.2011.10.045

5.	O’Connor C, Joffe H. Intercoder reliability in qualitative research: Debates and practical guidelines. International Journal of Qualitative Methods. 2020;19:1609406919899220. doi: 10.1177/1609406919899220

6.	Nowell LS, Norris JM, White DE, Moules NJ. Thematic Analysis:Striving to Meet the Trustworthiness Criteria. International Journal of Qualitative Methods. 2017;16(1):1609406917733847. doi: 10.1177/1609406917733847

7.	Althubaiti A. Information bias in health research: definition, pitfalls, and adjustment methods. Journal of Multidisciplinary Healthcare. 2016;9:211-7. doi: 10.2147/jmdh.S104807

8.	Warne JP, Gruber AH. Transitioning to minimal footwear: A systematic review of methods and future clinical recommendations. Sports Medicine - Open. 2017;3(1):33. doi: 10.1186/s40798-017-0096-x

9.	Hébert-Losier K, Knighton H, Finlayson SJ, Dubois B, Esculier J-F, Beaven CM. Biomechanics and subjective measures of recreational male runners in three shoes running outdoors: A randomised crossover study. Footwear Science. 2024;16(1):13-23. doi: 10.1080/19424280.2023.2283460

10.	Fisher RJ. Social desirability bias and the validity of indirect questioning. Journal of Consumer Research. 1993;20(2):303-15. doi: 10.1086/209351

11.	Bispo Júnior JP. Social desirability bias in qualitative health research. Revista de Saúde Pública. 2022;56:101. doi: 10.11606/s1518-8787.2022056004164

12.	Sung B, Vanman E, Hartley N. Interest, but not liking, drives consumer preference toward novelty. Australasian Marketing Journal. 2019;27(4):242-8. doi: 10.1016/j.ausmj.2019.06.003

13.	Reber R, Schwarz N, Winkielman P. Processing fluency and aesthetic pleasure: Is beauty in the perceiver's processing experience? Personality and Social Psychology Review. 2004;8(4):364-82. doi: 10.1207/s15327957pspr0804_3

14.	Hirschman EC. Innovativeness, Novelty Seeking, and Consumer Creativity. Journal of Consumer Research. 1980;7(3):283-95. doi: 10.1086/208816

15.	Zajonc RB. Mere exposure: A gateway to the subliminal. Current Directions in Psychological Science. 2001;10(6):224-8. doi: 10.1111/1467-8721.00154

16.	Crowell HP, Davis IS. Gait retraining to reduce lower extremity loading in runners. Clinical Biomechanics. 2011;26(1):78-83. doi: 10.1016/j.clinbiomech.2010.09.003
