# Supplementary material for: How does it feel to run in minimalist and advanced footwear technology shoes: A qualitative study involving male recreational runners
Source: PLoS One. 2025 Dec 26;20(12):e0338743. doi: 10.1371/journal.pone.0338743 (PMC12742779; doi:10.1371/journal.pone.0338743)
Supplement: Attachment — Submitted filename: PONE_Response_R2.docx [file pone.0338743.s005.docx]

**Manuscript Number**: PONE-D-25-14476R1

**Title:** How does it feel to run in minimalist and advanced footwear technology shoes: A mixed-methods study involving male recreational runners

**Journal:** PLOS ONE

Thank you to the peer-reviewers for reconsidering the suitability of our work for publication. We are pleased to have satisfied the comments of the initial reviewers. We believe these changes have resulted in a substantially improved manuscript. We are grateful for the opportunity to further clarify aspects of our work with the help of the comments from an additional expert reviewer.

As requested, we have revised our manuscript according to the comments received. We provide below point-by-point responses to all comments. To facilitate the review process, changes to the original manuscript are formatted in red font except for areas where we have removed text. We trust that the reviewers and editorial board find our responses meet their expectations.

**Journal Requirements:**

If the reviewer comments include a recommendation to cite specific previously published works, please review and evaluate these publications to determine whether they are relevant and should be cited. There is no requirement to cite these works unless the editor has indicated otherwise. 

**Response**: Noted with thanks.

Please review your reference list to ensure that it is complete and correct. If you have cited papers that have been retracted, please include the rationale for doing so in the manuscript text, or remove these references and replace them with relevant current references. Any changes to the reference list should be mentioned in the rebuttal letter that accompanies your revised manuscript. If you need to cite a retracted article, indicate the article’s retracted status in the References list and also include a citation and full reference for the retraction notice.

**Response**: Noted with thanks. Below are the changes to the reference list.

Addition of: Hannigan JJ, Westley L, Jin L. Injury and performance-related running biomechanics in advanced footwear technology compared to minimalist footwear. Footwear Sci. 2024;16:115-121. doi: 10.1080/19424280.2024.2327317

Reviewers' comments:

Reviewer's Responses to Questions

**Comments to the Author**

1. If the authors have adequately addressed your comments raised in a previous round of review and you feel that this manuscript is now acceptable for publication, you may indicate that here to bypass the “Comments to the Author” section, enter your conflict of interest statement in the “Confidential to Editor” section, and submit your "Accept" recommendation.

Reviewer #1: All comments have been addressed

Reviewer #3: All comments have been addressed

Reviewer #4: (No Response)

2. Is the manuscript technically sound, and do the data support the conclusions?

The manuscript must describe a technically sound piece of scientific research with data that supports the conclusions. Experiments must have been conducted rigorously, with appropriate controls, replication, and sample sizes. The conclusions must be drawn appropriately based on the data presented.

Reviewer #1: Yes

Reviewer #3: Yes

Reviewer #4: Yes

3. Has the statistical analysis been performed appropriately and rigorously?

Reviewer #1: Yes

Reviewer #3: Yes

Reviewer #4: N/A

4. Have the authors made all data underlying the findings in their manuscript fully available?

The [PLOS Data policy](http://track.editorialmanager.com/CL0/http:%2F%2Fwww.plosone.org%2Fstatic%2Fpolicies.action%23sharing/1/010f019a6b98ed3c-9a13349b-af08-4ce5-abe9-ba81a2ecd7c4-000000/w6dfH55zHdV5C0wMt19r_B0vzPzNjl0j7wYDxc87kx4=236) requires authors to make all data underlying the findings described in their manuscript fully available without restriction, with rare exception (please refer to the Data Availability Statement in the manuscript PDF file). The data should be provided as part of the manuscript or its supporting information, or deposited to a public repository. For example, in addition to summary statistics, the data points behind means, medians and variance measures should be available. If there are restrictions on publicly sharing data—e.g. participant privacy or use of data from a third party—those must be specified.

Reviewer #1: Yes

Reviewer #3: Yes

Reviewer #4: Yes

5. Is the manuscript presented in an intelligible fashion and written in standard English?

PLOS ONE does not copyedit accepted manuscripts, so the language in submitted articles must be clear, correct, and unambiguous. Any typographical or grammatical errors should be corrected at revision, so please note any specific errors here.

Reviewer #1: Yes

Reviewer #3: Yes

Reviewer #4: Yes

6. Review Comments to the Author

Please use the space provided to explain your answers to the questions above. You may also include additional comments for the author, including concerns about dual publication, research ethics, or publication ethics. (Please upload your review as an attachment if it exceeds 20,000 characters)

**Reviewer #1:** The authors have answered the comments of the reviewers. I have considered the article almost good in its initial version, so now it is adequate for publishing.

**Response:** Thank you for your time and thoughtful consideration of our work.

**Reviewer #3:** (No Response)

**Reviewer #4:** The manuscript presents a well-designed and engaging qualitative exploration of runners’ perceptions of minimalist and advanced footwear technology (AFT) shoes. The topic is timely and contributes meaningfully to the growing body of literature on subjective footwear experiences. It appears my review is after one round of peer reviews already occurred. Thus, I only have 1 major and a few minor concerns to address.

**Response:** Thank you for your time and overall positive review of our work.

Major Concern

1. Study Design Classification
This study is primarily qualitative, with limited quantitative data (e.g., rankings, run times). As currently described, it is not clear that the study meets the criteria for a mixed-methods design. Please justify the “mixed-methods” terminology or modify the study design description to be qualitative.

**Response:** We thank you for raising this point. Given that the qualitative aspect of the work drove our analysis and interpretation, we agree that reclassifying our work as being “a qualitative study” is appropriate. We have therefore updated our manuscript accordingly.

***Title****: How does it feel to run in minimalist and advanced footwear technology shoes: A qualitative study involving male recreational runners*

Minor Concerns

Introduction

Third paragraph
-While it did not employ a Delphi approach, I consider Frederick (2022, Footwear Science, “Let’s Just Call it Advanced Footwear Technology”) to be the accepted definition for AFT. I recommend citing this paper as the field’s definitional reference.

**Response:** We have altered the wording of this section to incorporate the definition Frederick proposed in his editorial piece, but feel it is important to state that this is a not a true consensus.

***Introduction****: In contrast, experts propose AFT defines a “performance-enhancing footwear technology that combines lightweight, resilient midsole foams with rigid moderators and pronounced rocker profiles in the sole.” [1]. Although there is no true consensus definition for AFT shoes [2], they typically feature a thick midsole and stiff curved element that increases longitudinal bending stiffness [2], with their overall design aimed at reducing running energy costs and enhance performance.*

-When discussing instability in AFT, consider also citing Hannigan et al. (2024, “Injury and performance-related running biomechanics in advanced footwear technology compared to minimalist footwear”), which demonstrated greater eversion excursion in AFT compared to minimalist footwear, supporting your claim. Citing Tenforde et al. (2023) in this section would also be warranted, as it is cited later but conceptually aligns here.

**Response**: Thank you for highlighting this work, as the greater eversion in AFT is relevant to the perceived instability of runners. As Tenforde is cited a few lines above where we have added Hannigan, we decided not to cite Tendorde again in this instance.

***Discussion:*** *The concerns expressed from runners regarding ankle sprains in turns in AFT are noteworthy, pointing toward compromised frontal plane stability [3] and increased ankle eversion excursion [4] linked with their design.*

Fourth paragraph
-Minor point, but most AFT could be considered maximalist, so stating they are similar to traditional shoes (which generally lack a carbon plate, lack highly compliant foam, and have lower stack heights) appears a bit misleading

**Response**: We appreciate this perspective and comment. Whilst we agree that AFT would be more comparable to maximal shoes than minimal shoes, a limiting factor is the absence of a consensus definition on maximal shoes [5]. Therefore, we have aimed to take a balanced approach when rewording this sentence by acknowledging that AFT are more similar to both traditional and maximal shoes compared with minimalist shoes.

***Introduction:*** Despite their novel features, AFT shoes are more similar in design to traditional and maximal running shoes than minimalist ones,…

Fifth paragraph
-If possible, add a brief note explaining why only male runners were recruited, as this will help contextualize the study’s purpose and generalizability.

**Response**: We have added a sentence in the participant section to highlight why only male runners were targeted. As the participant section is the sixth paragraph, we did not add this statement at both locations to avoid repetition.

***Materials and methods****: Male runners were recruited due to the shoe availability at the time of the study and for sampling convenience.*

Methods

-Please explain whether the male-only design was chosen to limit variability (e.g., controlling for sex-based biomechanical differences) or due to recruitment convenience.

**Response**: Thanks for this comment, which has been addressed in-text as described in response immediately above.

-Line 102 – “the running of the study” likely refers to the study’s conduct, not literal running alongside participants. Consider alternative wording such as “the conduct of the study” to avoid confusion.

**Response**: This is a valid point. We have changed the term to “in the conduct of the study”, as suggested.

-Line 123 – The description of your qualitative approach includes multiple philosophical terms (“interpretivist epistemology,” “constructivist ontology,” “neo-positivist methods”). While technically correct, the paragraph may be unnecessarily dense. The final sentence emphasizing your phenomenological approach conveys the key point effectively. Consider simplifying or consolidating this section for clarity.

**Response**: While we fundamentally agree, the previous round of reviews led to expansion of our description of the qualitative approach. Therefore, we have decided to retain the current level of detail regarding the methodological approach. To improve the clarity of this section, we have made several minor grammatical changes, which we hope will make this section appear less dense for readers.

“This study primarily follows an interpretivist epistemology with a constructivist ontology, recognising that runners’ perceptions of footwear are shaped by individual experiences. However, data collection was conducted using a neutral, structured interview approach inspired by neo-positivist methods to minimise interviewer bias. Qualitative data analysis was conducted using an interpretive phenomenological approach, aligning with the constructivist assumption that meaning is co-constructed through lived experience.”

-Please confirm whether participants wearing the Garmin 245 Music watches were not listening to music during the trials, as this could otherwise influence perceived comfort or performance.

**Response**: Thanks for raising this. We now clearly specify that the watch was worn “without listening to music”.

Results / Quotations

-Several participant quotes contain minor grammatical or spelling errors. Because these were verbal interviews, please clarify your editorial policy on quote correction. If readability is affected, limited grammatical correction may be acceptable, provided meaning is preserved.
Line 218: “their” should be “they’re.”
Line 232: “the” should be “they are.”
Line 368: “your” should be “you’re.”

-Some participant quotes are in italics while some are in single quotation marks — should this be revised for consistency?

**Response:** Thank you to the reviewer for your careful attention to detail. We have reviewed participants quotes for spelling and grammatical errors and have made amendments where required. Regarding the presentation of participants quotes within quotation marks or in italicised text: quotation marks have been used throughout when a participant quote is embedded within a paragraph (to signpost that the text represents participant data) whereas supporting quotes which are positioned outside of paragraphs have been presented in italics. The typesetters can ultimately adjust as required.
Results

-Line 403: Please clarify whether participants changed their responses from “would not buy” to “would buy,” or the reverse, when describing shifts in purchasing decisions.

**Response:** We have added the following clarifying the Line 403, reminding readers that all changes (or no changes) in perceptions can be found in Fig 2.

***Results:*** *Only two runners expressed interest in purchasing the FLAT after running (one changing from ‘would not buy’ to ‘buy’, Fig 2),*

1.	Frederick EC. Let’s just call it advanced footwear technology (AFT). Footwear Science. 2022;14(3):131-. doi: 10.1080/19424280.2022.2127526

2.	Hébert-Losier K, Pamment M. Advancements in running shoe technology and their effects on running economy and performance – A current concepts overview. Sports Biomechanics. 2023;22(3):335-50. doi: 10.1080/14763141.2022.2110512

3.	Hoogkamer W. More isn’t always better. Footwear Science. 2020;12(2):75-7. doi: 10.1080/19424280.2019.1710579

4.	Hannigan JJ, Westley L, Jin L. Injury and performance-related running biomechanics in advanced footwear technology compared to minimalist footwear. Footwear Science. 2024;16(2):115-21. doi: 10.1080/19424280.2024.2327317

5.	Pollard CD, Ter Har JA, Hannigan JJ, Norcross MF. Influence of maximal running shoes on biomechanics before and after a 5K run. Orthopaedic Journal of Sports Medicine. 2018;6(6):2325967118775720. doi: 10.1177/2325967118775720
