# Supplementary material for: How does it feel to run in minimalist and advanced footwear technology shoes: A qualitative study involving male recreational runners
Source: PLoS One. 2025 Dec 26;20(12):e0338743. doi: 10.1371/journal.pone.0338743 (PMC12742779; doi:10.1371/journal.pone.0338743)
Supplement: Attachment — Submitted filename: PONE_Response_R3.docx [file pone.0338743.s006.docx]

**Manuscript Number**: PONE-D-25-14476R2

**Title:** How does it feel to run in minimalist and advanced footwear technology shoes: A qualitative study involving male recreational runners

**Journal:** PLOS ONE

==============================

**Dear Authors,**

Your manuscript has been accepted for publication, pending minor revisions.

Please address the comment provided by Reviewer 4 and resubmit the revised manuscript.

==============================

We are pleased with the decision of the editorial board to accept our manuscript pending addressing the comment provided by Reviewer 4.

**Reviewer #4:** All comments were addressed and the paper is ready for publication.

I'll add one minor note. It appears the authors adjusted the discussion section in response to my comment below, while my comment was instead directed at the introduction section. I'll leave it to the authors whether to make any further edits to the introduction section, as the edits made did strengthen the discussion section.

Regarding my comment: "When discussing instability in AFT, consider also citing Hannigan et al. (2024, “Injury and performance-related running biomechanics in advanced footwear technology compared to minimalist footwear”), which demonstrated greater eversion excursion in AFT compared to minimalist footwear, supporting your claim. Citing Tenforde et al. (2023) in this section would also be warranted, as it is cited later but conceptually aligns here.

Response: We thank you for the clarification. We have integrated the two references (which were already cited in our previous version) in the introduction where we discuss injury and stability.

***Introduction:*** *However, their rapid adoption raises injury concerns [1, 2], as features like increased stack height may compromise stability [3], potentially leading to injuries such as ankle sprains especially during sharp turns in races [4].*

**References**

1.	Hébert-Losier K, Pamment M. Advancements in running shoe technology and their effects on running economy and performance – A current concepts overview. Sports Biomechanics. 2023;22(3):335-50. doi: 10.1080/14763141.2022.2110512

2.	Tenforde A, Hoenig T, Saxena A, Hollander K. Bone stress injuries in runners using carbon fiber plate footwear. Sports Medicine. 2023;53(8):1499-505. doi: 10.1007/s40279-023-01818-z

3.	Hannigan JJ, Westley L, Jin L. Injury and performance-related running biomechanics in advanced footwear technology compared to minimalist footwear. Footwear Science. 2024;16(2):115-21. doi: 10.1080/19424280.2024.2327317

4.	Hoogkamer W. More isn’t always better. Footwear Science. 2020;12(2):75-7. doi: 10.1080/19424280.2019.1710579
