# Supplementary material for: How does it feel to run in minimalist and advanced footwear technology shoes: A qualitative study involving male recreational runners
Source: PLoS One. 2025 Dec 26;20(12):e0338743. doi: 10.1371/journal.pone.0338743 (PMC12742779; doi:10.1371/journal.pone.0338743)
Supplement: Attachment — Submitted filename: PONE-D-25-14476 reviewer comments.docx [file pone.0338743.s002.docx]

This study employed a qualitative approach to explore male recreational runners' subjective experiences with minimalist running shoes (FLAT) and advanced footwear technology (AFT) shoes, revealing complex perceptions regarding comfort, performance, biomechanical changes, and injury risk. The findings indicate that runners' evaluations of footwear are a dynamic, iterative process influenced by novelty, familiarity, and actual running experience. OWN shoes were preferred due to familiarity, while AFT and FLAT elicited mixed reactions due to their extreme designs. The study highlights the need for runners to balance comfort, performance, and injury risk when selecting footwear and recommends a gradual transition to new shoe models. However, the research has limitations, including a restricted sample, limited shoe variety, and methodological constraints in data collection. Detailed comments from the reviewer are as follows:

1.Limited sample diversity. Recommend including female runners or explicitly stating conclusions apply only to male populations.

2.Restricted shoe representation. Testing only one AFT and FLAT model limits generalizability. Suggest adding other models within each category to enhance external validity.

3.Insufficient running distance. 1.5 km may not adequately reflect long-term adaptation. Recommend extending single-run distance or adding multiple trials.

4.Unclear pace control. "Comfortable pace" and "race pace" lack quantitative definitions. Should supplement with pace ranges or heart rate/RPE data to standardize intensity.

5.Lack of objective measures. Over-reliance on subjective interviews. Recommend incorporating biomechanical (e.g., gait analysis) or physiological (e.g., metabolic cost) data to validate perceptions.

6.Interview method limitations. Neo-positivist approach may constrain depth. Suggest semi-structured or phenomenological interviews for richer subjective data capture.

7.Inadequate controls for order effects. Using OWN shoes consistently as baseline may introduce bias. Recommend full randomization of all shoe testing sequences.

8.Unstandardized environmental factors. Outdoor trials affected by weather/terrain. Should document conditions (temperature, surface) and discuss potential impacts.

9.Insufficient data analysis transparency. Need detailed description of inter-coder reliability checks (e.g., Kappa values) in thematic analysis to enhance credibility.

10.Missing participant validation. Recommend member checking by returning preliminary findings to participants to ensure interpretive accuracy.

11.Unreported blinding effectiveness. Despite painted shoes, should assess if participants identified brands/types through other cues.

12.Long-term adaptations unexplored. Single trial cannot reflect prolonged use effects. Recommend adding runners' perspectives on transition periods or extended usage.
